# The enigmatic monotypic crab plover *Dromas ardeola* is closely related to pratincoles and coursers (Aves, Charadriiformes, Glareolidae)

**DOI:** 10.1590/S1415-47572010000300033

**Published:** 2010-09-01

**Authors:** Sergio L. Pereira, Allan J. Baker

**Affiliations:** 1 Department of Natural History, Royal Ontario Museum, Toronto, ONCanada; 2 Department of Ecology and Evolution, University of Toronto, TorontoCanada

**Keywords:** *Dromas*, Charadriiformes, Bayesian tree inference, phylogenetics, systematics

## Abstract

The phylogenetic placement of the monotypic crab plover *Dromasardeola* (Aves, Charadriiformes) remains controversial. Phylogenetic analysis of anatomical and behavioral traits using phenetic and cladistic methods of tree inference have resulted in conflicting tree topologies, suggesting a close association of *Dromas* to members of different suborders and lineages within Charadriiformes. Here, we revisited the issue by applying Bayesian and parsimony methods of tree inference to 2,012 anatomical and 5,183 molecular characters to a set of 22 shorebird genera (including *Turnix*). Our results suggest that Bayesian analysis of anatomical characters does not resolve the phylogenetic relationship of shorebirds with strong statistical support. In contrast, Bayesian and parsimony tree inference from molecular data provided much stronger support for the phylogenetic relationships within shorebirds, and support a sister relationship of *Dromas* to Glareolidae (pratincoles and coursers), in agreement with previously published DNA-DNA hybridization studies.

The monotypic crab plover *Dromas ardeola* (Aves, Charadriiformes, Dromadidae) is very unusual among shorebirds regarding many anatomical and behavioral traits (Rands, 1996). Hence, it is not surprising that its phylogenetic affinities are not well established with these characters. For example, three studies using the same set of osteological characters, but differing in the method of analysis and character coding, have recovered conflicting phylogenies that placed *Dromas* plus several members of the suborders Charadrii and Lari within an unresolved clade (Strauch, 1978), or as a sister lineage to a clade containing Glareolidae plus Burhinidae embedded within the former family (Mickevich and Parenti, 1980), or yet as a sister lineage to all Lari (Chu, 1995). Based on non-cladistic analyses, skeletal and morphological similarities suggested that *Dromas* may be closely related to thick-knees (Charadrii, Burhinidae), while plumage characters placed it closely related to avocets (Charadrii, Recurvirostridae), and burrow-nesting behavior linked it to auks (Lari, Alcidae) (reviewed in Rands, 1996; Sibley and Ahlquist, 1990). A recent cladistic analysis of an extensive anatomical data set of birds did not recover the monophyly of any of the three suborders within Charadriiformes, and placed *Dromas* as a sister lineage to some members of Scolopaci plus a clade containing Lari and Charadrii, but excluding jacanas (Scolopaci, Jacanidae) (Livezey and Zusi, 2007). From a molecular perspective, the phylogenetic affinities of the crab plover has only been studied under a phenetic approach using DNA-DNA hybridization experiments (Sibley and Ahlquist, 1990), which suggested a closer relationship with coursers and pratincoles (Lari, Glareolidae).

To evaluate the phylogenetic affinities of the crab plover *Dromas ardeola*, we performed a Bayesian phylogenetic analysis in a taxonomic subset of 2,021 anatomical characters previously published for birds (Livezey and Zusi, 2006). Taxa included in the subset (Table 1) were those for which there are DNA sequences for the same species or a congeneric species (Baker *et al.*, 2007). The analysis was performed in MrBayes 3.1 (Ronquist and Huelsenbeck, 2003) using the Mk model of evolution. We set the command *lset coding* = *all rates* = *invgamma* to account for the inclusion of 1,210 invariable anatomical characters and avoid overestimation of branch lengths (Lewis, 2001). Two independent runs were performed in parallel for 2 million generations. Trees were samples in every thousand generations, and the first 201 trees were discarded after checking for convergence of algorithm.

We amplified and sequenced the nuclear RAG-1, and mitochondrial small ribosomal subunit (12S rDNA), cytochrome *b* (cyt *b*) and NADH dehydrogenase subunit 2 (ND2) genes for two crab plover specimens, following published primers and protocols (Pereira and Baker, 2004). Both L- and H-strands sequences were checked for ambiguities and a consensus sequence was created for each gene in Sequencher 4.1.2 (GeneCodes, Ann Arbor, Michigan). Consensus sequences were aligned visually in MacClade 4.0 (Maddison and Maddison, 2000). No variation was found between the two specimens, except for a third position transition in cyt *b*. All sequences obtained in this study were deposited in GenBank (accession numbers HM369458 to HM369458). Ambiguously aligned regions for the 12S rDNA were excluded from the analysis. The aligned molecular data set of 5,183 nucleotides contains the same genera as in the anatomical data set. We inferred the molecular phylogenetic relationships in MrBayes 3 (Ronquist and Huelsenbeck, 2003), assuming that each gene evolves following a general time-reversible model of evolution (GTR), and accounting for gamma-distributed rate variation (G) and a proportion of invariable sites (I), as suggested by the Akaike Information Criterion implemented in Modeltest 3.7 (Posada and Crandall, 1998). A codon-based partitioned model was also applied, where each codon position of protein-coding genes and non-coding positions of 12S rDNA were allowed to evolve following the GTR+G+I model. Bayesian trees were sampled as described above for the anatomical data set. We also inferred tree topology using maximum parsimony through heuristic search (branch swap = TBR, nreps = 100), and estimate branch support with 1,000 heuristic bootstrap replicates in PAUP 4.0b10 (Swofford, 2001).

Anatomical and molecular data evolve at different rates over time and across lineages. The combined phylogenetic analysis of these characters (total-evidence approach) may provide support for different parts of the phylogenetic tree, and/or reveal hidden conflict that is highly supported by one but not both data sets (Pereira and Baker, 2005). We combined the anatomical and molecular data sets and performed a Bayesian tree inference using the models of evolution described above for each individual data set.

The Bayesian analysis of the anatomical data set performed here suggested that *Dromas* is a sister lineage to Haematopodidae, with Posterior Probability (PP) = 0.93 (Figure 1). Many nodes have PP < 0.95, which are considered weakly supported, and the PP of the consensus tree among 88 trees present in the 95% credible interval is 0.23. The consensus Bayesian tree obtained here is considerably different from the maximum parsimony topology derived from more inclusive taxon data set (Livezey and Zusi, 2007). The parsimony tree in Livezey and Zusi (2007) did not have strongly supported nodes among most shorebirds, did not recover the three Charadriiformes suborders as monophyletic, and placed Jacanidae followed by *Dromas* as sister groups to the remaining shorebirds (Livezey and Zusi, 2007).

The consensus Bayesian tree inferred from the molecular data set including the same genera as in the anatomical data set (Figure 2) placed *Dromas* as a sister lineage to Glareolidae with posterior probability (PP) = 0.95, in agreement with DNA-DNA hybridization studies (Sibley and Ahlquist, 1990). PP of the consensus molecular tree among 42 topologies in the 95% credible interval of trees is 0.29. The relationships among the remaining taxa were identical to those of our previous study, in which *Dromas* was not sampled (Baker *et al.*, 2007), except that *Rissa* and *Rynchopus* were placed as sister genera. The codon partitioned model and the parsimony tree topology was similar to that of Figure 2, except that *Chlidonias* and *Rissa* are sister genera, in exclusion of *Rynchopus* (PP = 0.68; bootstrap support = 74%), in agreement with our previous phylogeny including 90 Charadriiformes genera (Baker *et al.*, 2007).

The inferred Bayesian topology derived from the total-evidence approach (Figure S1) was identical to the topology obtained from the molecular data set (Figure 2) with two exceptions: (1) the position of *Turnix* was similar to the topology derived from the anatomical data alone (Figure 1), with PP = 0.98; and (2) *Dromas* was inferred to be a sister lineage to a clade including *Uria*, *Stercorarius*, *Rhyncops*, *Chlidonias* and *Rissa* (PP = 0.90), as opposed to a sister lineage to Glareolidae as inferred by the molecular data set (Figure 1). Hence, the conflicting and poorly supported topologies recovered in the analyses of the anatomical data set using two distinct methods of tree inference support our previous suggestion that anatomical characters cannot confidently resolve the phylogenetic relationships among shorebirds (Pereira and Baker, 2005). In fact, retention of ancestral polymorphism or parallel evolution in phylogenetically independent lineages caused by ecological, behavioral and/or physiological constraints seems to obscure the evolutionary history of many organisms (Pereira and Baker, 2005).

In conclusion, based on molecular sequence (this study) and DNA-DNA hybridization data (Sibley and Ahlquist, 1990), the crab plover *Dromasardeola* is sister group to pratincoles and coursers, as supported by Bayesian and parsimony analyses of DNA sequences of RAG-1, 12S rDNA, cyt b and ND2.

**Figure 1:**
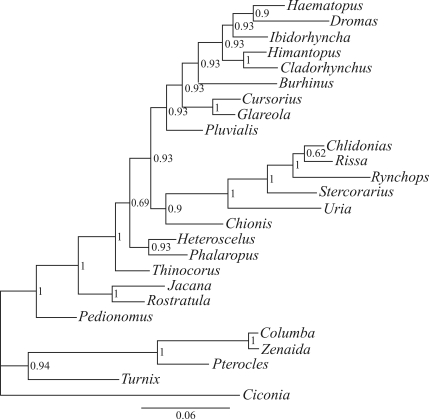
Consensus Bayesian tree derived from the anatomical data set. Numbers at nodes are posterior probabilities.

**Figure 2:**
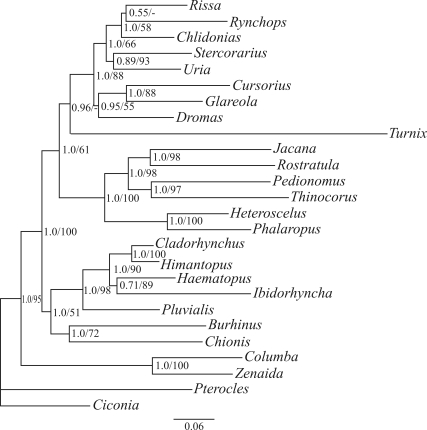
Consensus Bayesian tree derived from the molecular data set. Numbers at nodes are posterior probabilities /parsimony bootstrap percent proportions.

## Supplementary Material

The following online material is available for this article:

Figure S1 Consensus Bayesian tree derived from the total evidence approach.

This material is available as part of the online article from http://www.scielo.br/gmb

## Figures and Tables

**Table 1:** Taxon sampling and GenBank accession numbers.

Family	Species	RAG-1	12S rDNA	ND2	cyt *b*
Alcidae	*Uria lomvia*	EF373216	AJ242687	EF373273	U37308
Burhinidae	*Burhinus vermiculatus*	AY228771	EF380264	EF380265	-
Charadriidae	*Pluvialis squatarola*	EF373202	EF373101	EF373259	EF373151
Chionidae	*Chionis minor*	AY228782	DQ385272	DQ385085	DQ385221
Dromadidae	*Dromas ardeola*	HM369459	HM369462	HM369460	HM369461
Glareolidae	*Cursorius temminckii*	AY228780	DQ385277	DQ385090	DQ385226
	*Glareolamaldivarus*	-	EF373083	EF373241	EF373133
	*Glareola nuchalis*	AY228798	-	-	-
Haematopodidae	*Haematopus ater*	AY228794	NC_003713	NC_003713	NC_003713
Ibidorhynchidae	*Ibidoryncha struthersii*	EF373188	EF373086	EF373244	EF373136
Jacanidae	*Jacana jacana*	AY228776	DQ385273	DQ385086	DQ385222
Laridae	*Rissa tridactyla*	AY228785	DQ385280	DQ385093	DQ385229
Pedionomidae	*Pedionomus torquatus*	AY228789	DQ385276	DQ385089	DQ385225
Recurvirostridae	*Cladorhynchus leucocephalus*	EF373176	EF373074	EF373232	EF373125
	*Himantopus mexicanus*	AY228795	DQ385268	DQ385081	DQ385217
Rostratulidae	*Rostratula benghalensis*	AY228801	EF373107	EF373265	EF373156
Rynchopidae	*Rynchopsniger*	AY228784	DQ385281	DQ385094	DQ385230
Scolopacidae	*Heteroscelus incanus*	AY894213	AY894145	AY894179	AY894230
	*Phalaropus tricolor*	AY228778	AY894155	AY894189	AY894240
Stercoriidae	*Stercorarius longicaudus*	EF373208	EF373109	EF373267	EF373158
Sternidae	*Chlidonias leucoptera*	EF373175	EF373073	EF373231	EF373124
Thinocoridae	*Thinocorus rumicivorus*	EF373213	EF373112	EF373270	EF373160
Turnicidae	*Turnix sylvatica*	EF380262	DQ385283	DQ385096	DQ385232
Outgroup	*Pteroclesorientalis*	AY228767	-	-	-
	*Pterocles namaqua*	-	DQ385267	DQ385080	DQ385216
	*Columba livia*	EF373500	EF373295	AF353433	AF182694
	*Zenaida macroura*	EF373530	EF373325	EF373359	AF182703
	*Ciconia ciconia*	-	NC_002197	NC_002197	NC_002197
	*Ciconia abdimii*	HM369458	-	-	-
