# Supplementary material for: The enigmatic monotypic crab plover Dromas ardeola is closely related to pratincoles and coursers (Aves, Charadriiformes, Glareolidae)
Source: Genet Mol Biol. 2010 Sep 1;33(3):583–6. doi: 10.1590/S1415-47572010000300033 (PMC3036115; doi:10.1590/S1415-47572010000300033)
Supplement: Figure S1 — Consensus Bayesian tree derived from the total evidence approach. [file gmb-33-3-583-suppl1.pdf]

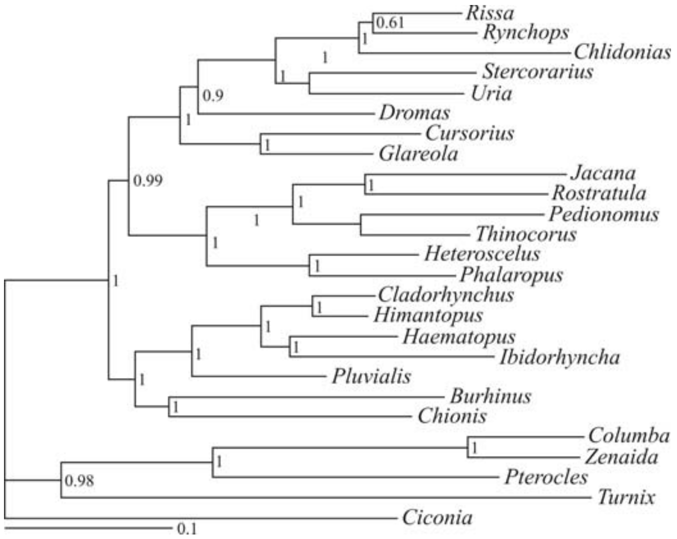

Figure S1 - Consensus Bayesian tree derived from the total evidence approach. Numbers at nodes are posterior probabilities.
